# From Molecules to the Clinic: Linking Schizophrenia and Metabolic Syndrome through Sphingolipids Metabolism

**DOI:** 10.3389/fnins.2016.00488

**Published:** 2016-11-08

**Authors:** Rolando I. Castillo, Leonel E. Rojo, Marcela Henriquez-Henriquez, Hernán Silva, Alejandro Maturana, María J. Villar, Manuel Fuentes, Pablo A. Gaspar

**Affiliations:** ^1^Translational Psychiatry Laboratory, Clínica Psiquiátrica Universitaria, Hospital Clínico Universidad de ChileSantiago, Chile; ^2^Departamento de Biología, Facultad de Química y Biología, Universidad de Santiago de ChileSantiago, Chile; ^3^Departamento de Laboratorios Clínicos, Escuela de Medicina, Pontificia Universidad Católica de ChileSantiago, Chile; ^4^Department of Pediatrics, Institute of Human Nutrition, College of Physicians and Surgeons, Columbia UniversityNew York, NY, USA; ^5^Department of Pathology and Cell Biology, Columbia UniversityNew York, NY, USA; ^6^Facultad de Medicina, Biomedical Neuroscience Institute, Universidad de ChileSantiago, Chile; ^7^Departamento de Psiquiatría, Clínica AlemanaSantiago, Chile

**Keywords:** metabolic syndrome, psychosis, schizophrenia, sphingolipids

## Abstract

Metabolic syndrome (MS) is a prevalent and severe comorbidity observed in schizophrenia (SZ). The exact nature of this association is controversial and very often accredited to the effects of psychotropic medications and disease-induced life-style modifications, such as inactive lifestyle, poor dietary choices, and smoking. However, drug therapy and disease-induced lifestyle factors are likely not the only factors contributing to the observed converging nature of these conditions, since an increased prevalence of MS is also observed in first episode and drug-naïve psychosis populations. MS and SZ share common intrinsic susceptibility factors and etiopathogenic mechanisms, which may change the way we approach clinical management of SZ patients. Among the most relevant common pathogenic pathways of SZ and MS are alterations in the sphingolipids (SLs) metabolism and SLs homeostasis. SLs have important structural functions as they participate in the formation of membrane “lipid rafts.” SLs also play physiological roles in cell differentiation, proliferation, and inflammatory processes, which might be part of MS/SZ common pathophysiological processes. In this article we review a plausible mechanism to explain the link between MS and SZ through a disruption in SL homeostasis. Additionally, we provide insights on how this hypothesis can lead to the developing of new diagnostic/therapeutic technologies for SZ patients.

## Introduction

Schizophrenia (SZ) is a severe and chronic psychotic disorder with a lifetime risk of 0.7% (Saha et al., 2005), in which genetic and environmental factors contribute to abnormal anatomical and functional connectivity of the brain (Stephan et al., 2006; Canu et al., 2015). Abnormal connectivity may be the underlying contributor to a wide range of symptoms observed in this disease, such as hallucinations, delusions, avolition, blunted affect, and cognitive dysfunctions (Gaspar et al., 2012). These symptoms often cause poor functional outcome, frequent co-morbidities, and increased mortality rate. For these reasons, SZ is ranked among the top 10 leading causes of disease-related disability in the world and is consistently demonstrated to have a major negative impact on quality of life (WHO, 2001).

The well-known association between SZ and metabolic syndrome (MS) may be a contributor to the poor long-term outcomes stated above. The prevalence of MS among medicated SZ patients is as high as 60%, compared to 30% in the general population (Malhotra et al., 2013; Papanastasiou, 2013) with the subsequent increment in cardiovascular morbi-mortality. The nature of this association has been frequently accredited to the adverse effects of psychotropic medications, especially second-generation antipsychotics (Jin et al., 2004; Peet, 2004; Filaković et al., 2012), and to disease-induced life-style modifications, such as inactive lifestyle, unhealthy diet and smoking (Peet, 2004). However, several studies have shown that increased insulin levels and glucose abnormalities can occur at early stages of the disease, in drug naïve first episode psychosis patients (Ryan et al., 2003; Spelman et al., 2007; Fernandez-Egea et al., 2009; Verma et al., 2009; Guest et al., 2010; Chadda et al., 2013) and in their first-degree relatives (Fernandez-Egea et al., 2008a,b). It is difficult to control for all the risk factors associated to MS (e.g., poor diet, lack of exercise, cigarette smoking, environmental stress, and drug abuse) in observational clinical studies of SZ patients, and indeed most studies often fail to properly control for all of these factors. However, the study of Fernandez-Egea et al., matched the groups by age, sex, heart rate, body mass index, tobacco consumption, and ethnic origin (Fernandez-Egea et al., 2009) demonstrating the plausibility of a predisposition for developing MS among SZ patients and their close relatives. This brings the testable hypothesis that MS in SZ patients would not only be linked to epigenetic factors, but also to genetic alterations, such as polymorphisms of enzymes and/or receptors involved in brain physiology (Yogaratnam et al., 2013).

Although there is evidence for the association of SZ with some MS associated genes like methylenetetrahydrofolate reductase (MTHFR) (Ellingrod et al., 2008) and alpha-1A adrenergic receptor (ADRA1A) (Cheng et al., 2012), none of these associations have led to a common metabolic pathway, which actually links MS with SZ providing the rationale for research toward a “drugable” therapeutic/prophylactic target. In this context, it is of utmost importance to identify common molecular pathways that explain this association in order to improve current diagnostic and therapeutic strategies and to avoid major metabolic complications.

Despite the fact that most of the current research on SZ has been focused on the role of synaptic proteins as etiopathogenic mediators of the disease (Stephan et al., 2009; Seshadri et al., 2013), the interest in membrane lipids (Horrobin, 1998), and specifically sphingolipids (SLs), has increased during the last decade (Schwarz et al., 2008; Narayan et al., 2009). Moreover, cumulative evidence suggests that SLs may link MS (Cowart, 2008) to psychotic disorders (Narayan and Thomas, 2011). Thus, SLs seemingly are interesting putative candidates for common etiopathogenic mediators of these disorders.

In this review we analyze the relevance of SLs in the MS associated with SZ, and we also highlight molecular events linking SZ with MS, in order to explain common etiopathogenic mechanisms and the predisposition of SZ patients to develop metabolic complications. Finally, according to our perspective, we propose a unifying, testable hypothesis to explain the participation of SLs in MS and SZ and also putative diagnostic/therapeutic applications, based on the detection of sub-families of SLs in peripheral samples of SZ patients.

## General aspects of sphingolipids

**Figure 1:**
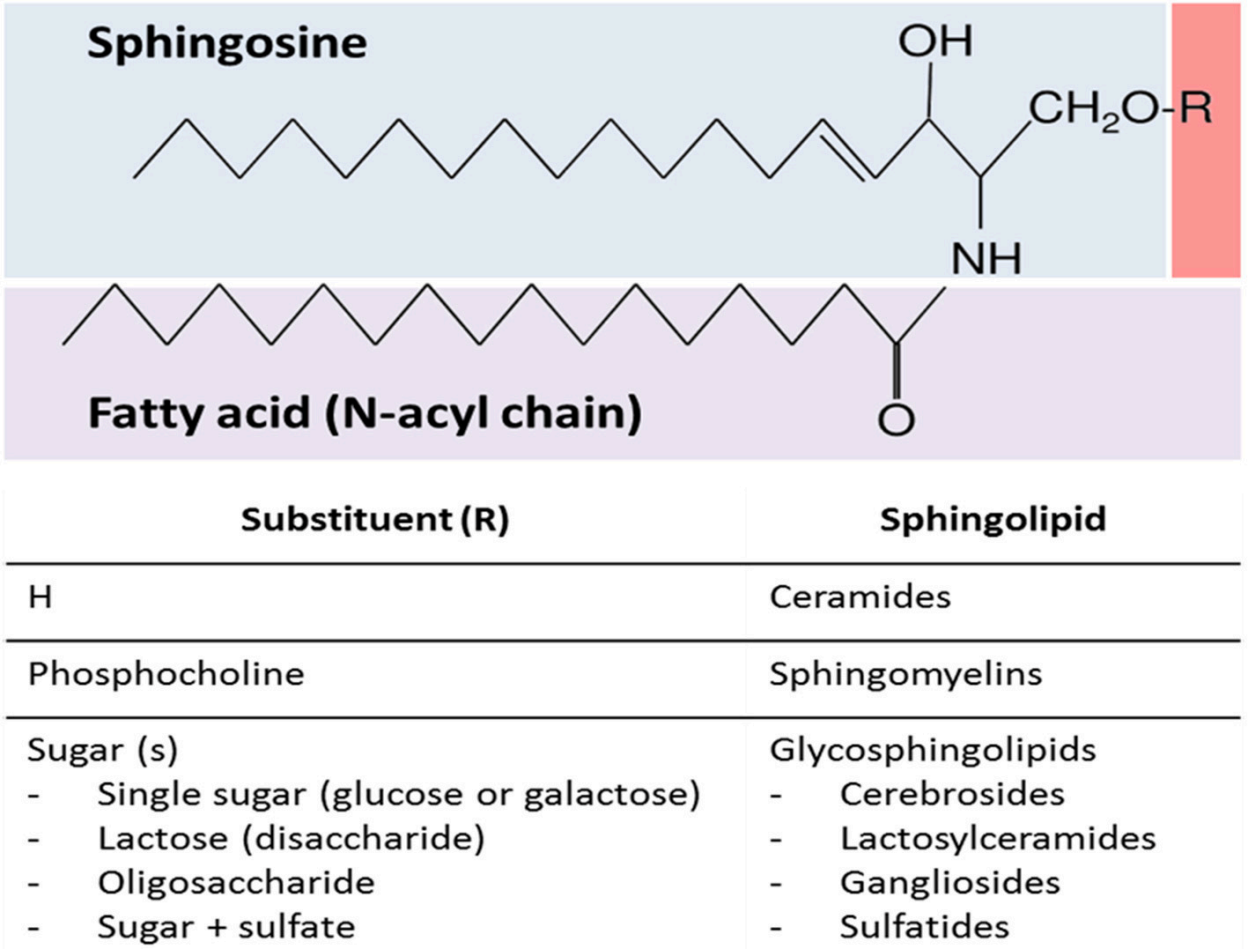
**General sphingolipid structure**. Sphingolipids are composed of a sphingosine backbone linked to a fatty acid via an amide bond. There are three main types of sphingolipids, which differ in their hydrophilic attachments: ceramides, sphingomyelins, and glycosphingolipids.

**Figure 2:**
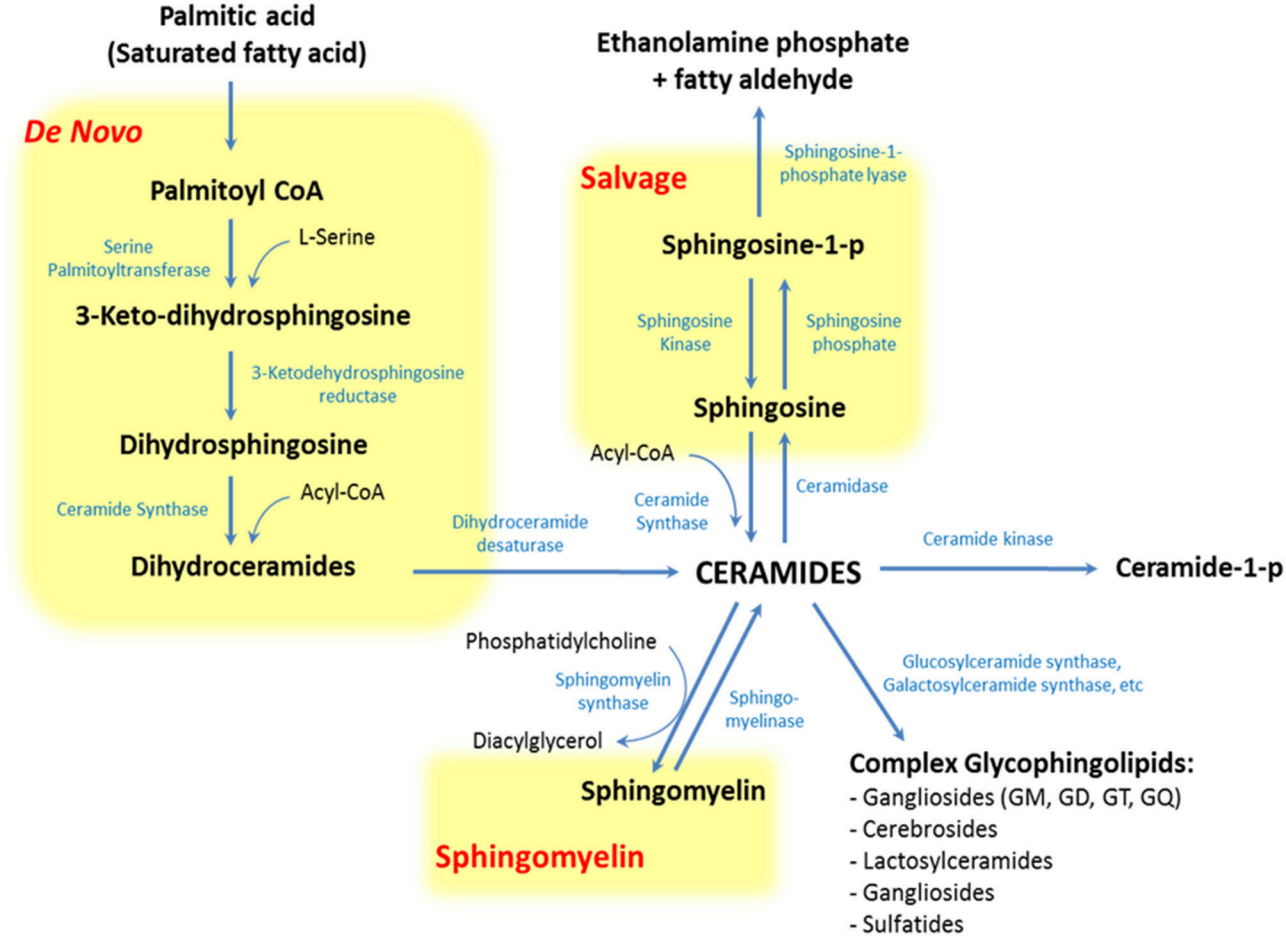
**Pathways of sphingolipid metabolism**. Sphingolipids have three major metabolic pathways: the *de novo* pathway coming from saturated fatty acids, the salvage pathway and the sphingomyelin pathway, all of which converge in ceramides.

SLs encompass a wide and complex family of membrane lipids in which a fatty acid is linked to a long sphingosine carbon backbone via an amide bond. Depending on their hydrophilic attachments, they can be divided into three main types of SLs: ceramides, sphingomyelins (SMs), and glycosphingolipids (GSLs) (Figure 1). The mammalian SLs metabolism has three major metabolic pathways: the *de novo* pathway coming from saturated fatty acids, the salvage pathway, and the SMs pathway, all of which converge in ceramides. The heterogeneous and complex GSLs are synthesized from ceramides (Figure 2). Due to their amphipathic properties, SLs are major components in cell membranes and are especially ubiquitous in the central nervous system (CNS) membrane “lipid rafts”(Colombaioni and Garcia-Gil, 2004; Aureli et al., 2015). Since their discovery in 1884, SLs have been shown to be involved in several neuropsychiatric (Kolter and Sandhoff, 2006; Adibhatla and Hatcher, 2008; Haughey, 2010), metabolic, immune, oncogenic, and inflammatory disorders (Lahari and Futerman, 2007; Zeidan and Hannun, 2007). Today it is widely accepted that the biophysical and signaling properties of SLs allow them to participate in the regulation of several key cellular processes, such as membrane microdomains organization, allosteric modulation of membrane proteins, and cell to cell interaction, differentiation, proliferation, inflammation, and apoptosis (Hannun and Obeid, 2008). In fact, SLs participate in several neuronal and metabolic processes, making them crucial to the normal and pathological functioning of these tissues.

## Metabolic syndrome and sphingolipids

### Metabolic syndrome pathophysiology

MS represents a cluster of cardiovascular risk factors including glucose intolerance, atherogenic dyslipidemia, and hypertension, leading to an increased risk of cardiovascular morbidity and mortality (Isomaa et al., 2001). MS shows a tight association with central (or visceral) fat mass, which is a good predictor of severity for cardiovascular complications compared to peripheral fat mass (Matsuzawa et al., 2011). This is because central fat mass is a major source of active biomolecules, like adipokines, cytokines, and free fatty acids (FFAs), which promote a chronic pathological status of inflammation and oxidative stress (Holland et al., 2007; Kaur, 2014). Several molecular pathways have been studied to understand the events linking central fat mass, FFAs and MS (Holland et al., 2007), but current data do not yet provide compelling answers. In this context, emerging evidence demonstrates that overnutrition induces SLs synthesis and turnover, which in turn “remodels” SLs profiles and their topological distribution.

A plausible explanation for this is that overnutrition and visceral obesity change FFAs profile, principally elevating levels of saturated fatty acids (SFAs) (Holland and Summers, 2008; Heilbronn et al., 2013; Kaur, 2014). These molecules promote ceramides synthesis through the production of palmitate-CoA and acyl-CoA, SFAs-derivatives involved in the *de novo* and salvage pathway of SLs metabolism, respectively (Figure 2). Palmitate-CoA is a substrate for palmitoyltransferase, the rate limiting enzyme of the *de novo* synthesis pathway of ceramides, and it stimulates palmitoyltransferase in a concentration-dependent manner. On the other hand, acyl-CoA can increase ceramides synthesis as a substrate of ceramides synthase (CerS) in the salvage pathway (Figure 2; Merrill et al., 1988). These metabolic changes increase ceramides levels, which in turn modify intracellular signaling and promote MS-associated conditions, like atherothrombotic status and glucose intolerance (Cowart, 2008).

### Sphingolipids and alterations of the glucose metabolism

The current literature shows that certain SLs can impair glucose metabolism in different organs, including pancreatic, skeletal muscle, and adipose tissues.

In pancreatic islets, lipid oversupply increases the ceramides β-cell induced apoptosis (or “lipoapoptosis”) (Shimabukuro et al., 1998b; Unger and Orci, 2002) In fact, pharmacological inhibitors of the ceramides biosynthesis ameliorate the lipotoxic effect on pancreatic β-cells (Shimabukuro et al., 1998a). Interestingly, ceramides also inhibit the expression of pro-insulin genes through the activation of c-Jun N-terminal kinases (JNK), an inhibitor of kappa B kinase beta (IκKβ) (Summers, 2006). Both mechanisms decrease pancreatic insulin secretion.

Ceramides also contribute to the glucose-resistance phenotype in the skeletal muscle. Here, ceramides impair glucose caption by the inhibition of the kinase B (Akt/PKB) insulin signaling pathway (Chavez et al., 2003). This is effect is achieved through protein phosphatase 2 (PP2A) (Zinda et al., 2001) or protein kinase C zeta (PKCζ) dependent mechanisms (Bourbon et al., 2002) and also by the inhibition of Akt translocation to the plasma membrane (Stratford et al., 2001). Notably, *in vivo* studies on insulin resistant obese patients showed increased ceramides levels in muscle biopsies (Adams et al., 2004; Straczkowski et al., 2007) and newly discovered evidence has identified C16:0-ceramide as the principal mediator of obesity-related insulin resistance (Hla and Kolesnick, 2014; Raichur et al., 2014; Turpin et al., 2014).

**Figure 3:**
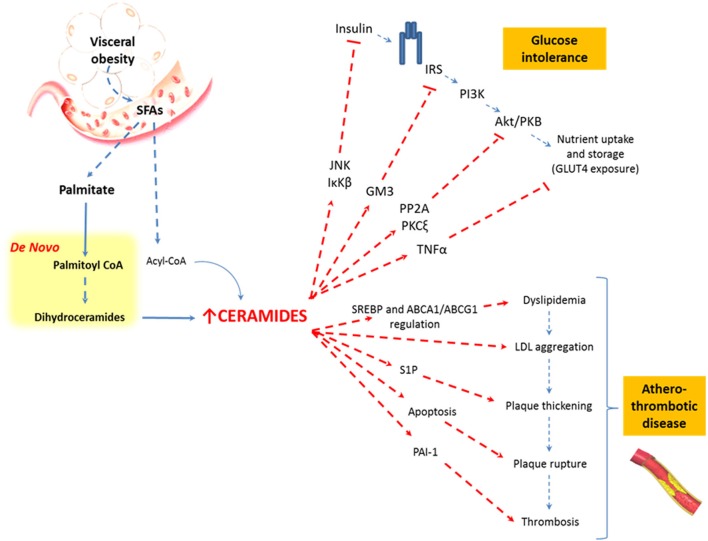
**Relationship between visceral obesity, sphingolipids, and metabolic abnormalities**. Saturated fatty acids are included in the sphingolipid pathways and are synthesized to ceramides. Ceramides affect the insulin signaling pathway and favor the atherothrombotic process through different metabolic pathways.

Finally, ceramides display other pro-inflammatory properties, inducing inflammation in adipose tissue via tumor necrosis factor α (TNFα) signaling (Bikman, 2012; Maceyka and Spiegel, 2014). TNFα induces insulin resistance by direct down-regulation of the insulin-regulatable glucose transporter type 4 (GLUT-4). TNFα also acts indirectly on the glucose uptake, by inducing the up-regulation of ganglioside GM3, which is a member of the SLs family that has proven to inhibit both: the insulin-mediated activation of insulin receptor substrate-1 (IRS-1) (Tagami et al., 2002) and the expression of insulin receptors from the lipid rafts (Kabayama et al., 2005). In sum, SLs can interrupt the insulin signaling pathway in at least four different forms (Figure 3).

### Sphingolipids and atherothrombosis

It is important to mention that SLs synthesis is necessary for the activation and subsequent regulation of the sterol regulatory element-binding proteins (SREBPs), which are key transcription factors in the lipid biosynthesis that regulate the transcription of enzymes involved in the synthesis of cholesterol, phospholipids, and fatty acids (Worgall et al., 2002; Worgall, 2008). Ceramides and SMs are also implicated in the regulation of cholesterol efflux by a mechanism that involves the cholesterol-efflux receptors ABCA1 and ABCG1. Thus, the ABCG1-mediated efflux of cholesterol is dependent on the cellular SM level in the plasma membrane, which implies that SM influences the high-density lipoproteins (HDL) plasma levels (Kaminski et al., 2006; Kobayashi et al., 2006; Sano et al., 2007).

Increased levels of ceramides seem to be related to the aggregation of low density lipoprotein (LDL) particles within the arterial wall, as aggregated LDL particles from atherosclerotic lesions (Guyton and Klemp, 1996) have 10- to 50-fold higher levels of ceramides than plasmatic LDL particles (Schissel et al., 1996). Moreover, ceramides have been shown to induce apoptosis of cells lining the vascular wall, a process implicated in plaque rupture of atherothrombotic disease (Mallat and Tedgui, 2001).

In parallel, S1P stimulates the proliferation of endothelial and smooth muscle cells in the vascular walls, promoting vascular remodeling and plaque thickening. Finally, ceramides and S1P increase plasminogen activator inhibitor-1 (PAI-1), an inhibitor of fibrinolysis (Soeda et al., 1995; Ito et al., 2013), favoring platelet activation and aggregation (Bhatia et al., 2004). Taken together, this evidence strongly suggests that SLs, and specifically ceramides, are crucial in the pathophysiology of the atherothrombotic process and cardiovascular events in obese, overfed, or malnourished patients (Figure 3).

Interestingly, obesity and alterations of the lipid metabolism have also been associated with psychotic disorders (Oresic, 2012). This topic will be reviewed in the next section.

## Healthy central nervous system and sphingolipids

Lipids in the brain account for 60% of its dry weight (Horrobin, 1998), and SLs in particular are found in high concentrations in the membranes of neurons and oligodendrocytes (van Echten-Deckert and Herget, 2006; Piccinini et al., 2010). This peculiar lipid composition and concentration favors the organization of laterally organized lipid-driven membrane domains. In fact, SLs have functional properties in inflammatory responses, and also structural properties as precursors of SMs and GSLs, which are necessary for the formation of the specialized membrane “lipid rafts” in neurons and oligodendrocytes myelin sheaths (Aureli et al., 2015). Lipid rafts are important for cell-to-cell interactions and modulation of membrane-associated proteins which determine normal synaptic neurotransmission (Allen et al., 2007) and axon-myelin stability and communication (Aureli et al., 2015). Due to these biologically critical features, SLs are a vital factor in the normal functioning of neurons and oligodendrocytes, determining appropriate axonal/synaptic connectivity, and neuronal survival (Posse de Chaves, 2006). In fact, the absence of the complex GSL synthesis function during early neurodevelopment stages is lethal in animal models (Jennemann et al., 2005).

## Schizophrenia pathophysiology

SZ is assumed to be a neurodevelopmental disorder in which genetic and environmental factors contribute to abnormal neuronal (Stephan et al., 2006; Kahn and Sommer, 2015) and glial cell (Hakak et al., 2001; Tkachev et al., 2003; Goudriaan et al., 2014; Bernstein et al., 2015) histology and function, leading to specific white matter and synaptic abnormalities that impair anatomical and functional brain connectivity.

Although the initial SZ pathognomonic events are unknown, a plausible inflammatory hypothesis states that environmental stress during early childhood (e.g., perinatal infections)—on top of increased genetic vulnerability—may stimulate the immune system to secrete pro-inflammatory cytokines contributing to subclinical but long-lasting neurotoxic processes. Current evidence supports the hypothesis that chronic inflammation impairs the monoaminergic and glutamatergic normal neurotransmission and damages white and gray matter structures in SZ patients (Müller et al., 2015; Najjar and Pearlman, 2015).

In fact, neuropathological studies in brains of SZ patients have shown that neuronal density increases, but glial density and dendritic spines decreases (Davis et al., 2003; Garver et al., 2008; Kyriakopoulos et al., 2008; Bakhshi and Chance, 2015; Gong et al., 2016). Other studies have also demonstrated that SZ brains display alterations in the expression of presynaptic proteins (e.g., increased SNARE interactions) and in the oligodendrocyte myelin sheet lamella (Bakhshi and Chance, 2015; Ramos-Miguel et al., 2015). There is also evidence of reduced brain weight and volume in a various brain regions, along with cerebral ventricular enlargement, loss of cerebral asymmetry, and reduction of fronto-temporal white matter tracts, all of which would impair structural connectivity of the brain (Stephan et al., 2006; Canu et al., 2015). Although there are other plausible hypotheses to explain the functional brain disconnection in SZ, we highlight a convergent hypothesis that explains the functional consequences of these heterogeneous synaptic and axonal anomalies based on the hypofunction of N-methyl-D-aspartate (NMDA) receptors in parvalbumin-positive γ-aminobutyric acid (GABA)-ergic interneurons. This abnormal NMDA signaling could lead to the disruption of the cortical tuning of glutamatergic pyramidal neurons. This hypofunction would cause a disengagement of the cortico-cortical and cortico-subcortical networks, impairing the functional connectivity of different brain areas (Ford et al., 2007; Gaspar et al., 2009, 2012; Uhlhaas and Singer, 2010; Gonzalez-Burgos and Lewis, 2012; Woodward et al., 2012; Cao et al., 2016).

Thus, inflammation and the loss of normal axonal or synaptic connectivity are seemingly responsible for the cognitive impairments and other classical symptoms (hallucinations, delusions, negative, and affective symptoms) observed in this disease, leading to the major social difficulties seen in these patients.

## Schizophrenia and sphingolipids

Numerous studies have shown alterations of the membrane lipid composition (Horrobin, 1998) and SLs metabolism in SZ (Yao et al., 2000; Schwarz et al., 2008; Narayan et al., 2009; Smesny et al., 2013) and other neuropsychiatric diseases (Posse de Chaves, 2006; Adibhatla and Hatcher, 2008; Jana et al., 2009; Kornhuber et al., 2009; Haughey, 2010; Mielke et al., 2010; Narayan and Thomas, 2011; Mühle et al., 2013; Saito and Saito, 2013). Due to their functional and structural roles, SLs could explain in part the inflammatory, synaptic and white matter changes that leads to disconnectivity in SZ (Jana et al., 2009; Please see Boxes 1, 2 for further discussion of SL abnormalities and their distribution across the central nervous system). Here we discuss how the clinical data and several model systems provide evidence on the abnormal metabolism of SLs and its close connections with neuroinflammation and disconnectivity in SZ.

Box 1 **Sphingolipid abnormalities in specific brain areas.**

Despite the fact that SLs-related neuropathological alterations have been linked to specific brain areas (e.g., prefrontal Cortex or thalamus), it is still difficult to reach coherent conclusions that can help to further understand this conundrum, if we are only based on the expression profiles of SLs-related enzymes. For instance, it is known that the six ceramide synthase (CerS) subtypes are differentially expressed through the nervous system. CerS determine the acyl-chain length of SLs, thus each tissue/cell has distinct SL acyl-chain length profiles. As an example, in the brain, CerS1 (which targets C18 acyl-chains) is distributed primarily in neurons, whereas CerS2, responsible for the synthesis of C22-C24 acyl-chain SLs, is expressed specifically in oligodendrocytes and Schwann cells. Several studies suggest that SLs with defined acyl-chain lengths play distinct pathophysiological roles in disease models (Park et al., 2014; Park and Park, 2015) and the alterations in the relative balance of these species might be of pathogenic relevance in SZ.

Box 2 **Sphingolipid abnormalities in the peripheral nervous system.**

If SZ patients suffer from a genetic SLs metabolism vulnerability, we should also expect peripheral neurologic abnormalities that go far beyond central nervous system dysfunctions. In fact, there is evidence of abnormal peripheral neuromuscular functioning in SZ patients, which supports the idea of SZ as a generalized disease rather than a specific central nervous system problem. For instance, there are alterations of the α-motoneuron excitability, increased motor unit fiber densities and increased branching of terminal motor nerves to elevated levels of muscular enzymes (Flyckt et al., 2000). Moreover, there is electrophysiological evidence on the functional consequences of these abnormalities showing impaired peripheral impulse propagation in electromyographic recordings (Borg et al., 1987). All of these findings are compatible with membrane defects.

### Sphingolipids and schizophrenia inflammatory hypothesis

Some SLs have pro-inflammatory properties (Maceyka and Spiegel, 2014) that can be involved in the initiation and maintenance of the subclinical inflammatory status linked to the above mentioned “neurodevelopmental vulnerability-stress-inflammatory model of SZ” (Müller et al., 2015). Moreover, SLs have also pro-apoptotic properties which suggest a plausible neurodegenerative component of SZ. Although SZ is not classified as a neurodegenerative disorder, it is clear that the progressive deterioration of some SZ subjects derives from incomplete apoptotic mechanisms at the local synaptic level (without inducing immediate neuronal death; Jarskog et al., 2005). Thus, SLs may be involved in both: pro-inflammatory and pro-apoptotic pathways.

In agreement with these plausible mechanisms, postmortem studies have found a significant decrease of phosphatidylcholine (PC) in the white matter of SZ patients (Yao et al., 2000). PC is the choline donor to SMs in neurons and oligodendrocytes. Therefore, low levels of PC are related to decreased SMs levels and, eventually, increased turnover of SMs to ceramides (Posse de Chaves, 2006). These findings may be associated with oligodendrocyte dysfunction, since SMs is a major component of myelin sheaths and ceramides have an apoptotic and inflammatory role.

Neuropathological evidence shows increased levels of phosphatidylserine in the left thalamic gray matter of SZ patients (Schmitt et al., 2004). This suggests the presence of a ceramides-mediated cell death, because phosphatidylserine acts as a potent activator of neutral sphingomyelinase (Sawai and Hannun, 1999) and stimulates phagocytosis of apoptotic cells (Chang et al., 2000).

**Figure 4:**
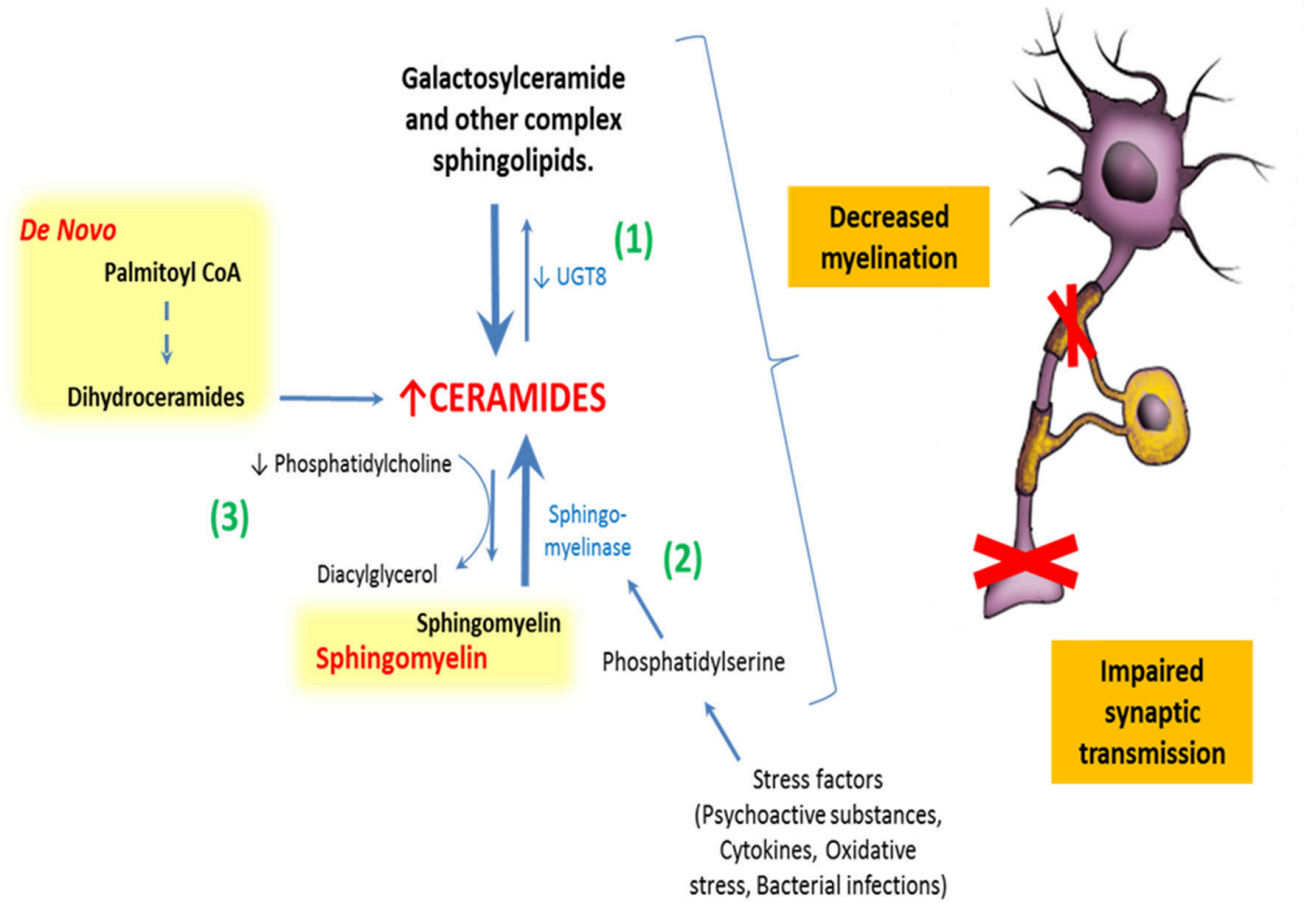
**Relationship between sphingolipids and schizophrenia**. There are at least three different described ways by which abnormal sphingolipid metabolism could impair normal neural functioning in humans: (1) By abnormal expression of galactosylceramide synthase increasing ceramides and decreasing galactosylceramides in myelin sheaths. (2) Stimulation of sphingomyelinase by stress factors, leading to a breakdown of sphingomyelins to ceramides. (3) And finally, by decreased synthesis of phosphatidylcholine. All of these mechanisms impact normal lipid membrane properties and might induce the synaptic and axonal disconnectivity seen in schizophrenia.

Consistent with the previous findings, bipolar disorder, and SZ patients are reported to show significantly increased levels of ceramides and decreased levels of PC in their white matter, regardless of antipsychotic treatment (Schwarz et al., 2008). Remarkably, environmental stress (such as psychoactive substances and oxidative stress) that are known to be involved in the development of several psychiatric disorders, also activate sphingomyelinase, increasing pro-inflammatory metabolites like ceramides and its derivatives (Posse de Chaves, 2006; Jana et al., 2009; Mühle et al., 2013) (Figure 4).

### Sphingolipids and the schizophrenia disconnectivity hypothesis

Genetic studies in humans and animals provide additional evidence for the role of abnormal SLs metabolism in the SZ disconnectivity hypothesis. Genes related to the metabolism of some structural SLs, such as SMs and GSLs, are altered in subjects with SZ, which might impair normal neuronal or glial function. The article of Narayan et al. is of particular interest in this context, as it shows decreased expression of UGT8-encoding for galactosyltransferase (CGT) and GAL3ST1–encoding for cerebroside sulfotransferase (CST) in these subjects. CGT is responsible for converting ceramides to galactosylceramides, and CST is responsible for the further metabolization of galactosylceramides to sulfatides. Diminished expression of these two genes may result in the overall decreased levels of galactosylceramides and sulfatides, which are major lipid components of the oligodendrocyte myelin sheath (Narayan et al., 2009) (Figure 4).

Interestingly, investigations with animal models confirm the *in vivo* consequences of these GSL pathologies. In fact, mice lacking CGT form unstable and functionally affected myelin and exhibit progressive demyelination, accompanied by severe motor coordination deficits (Zöller et al., 2005). Another study shows that the administration of GM1 ganglioside fully restored the mice's MK801-SZ cognitive model, correcting cognitive deficits and the impaired BDNF signaling (Ni et al., 2016). Finally, there is evidence that the induction of catalytically inactive SLs enzymes induces cognitive and behavioral abnormalities. For example, some inactive CerS-specific subtypes induce myelin sheath GSLs and glycoprotein defects, as well as behavioral abnormalities that include motor, exploration and habituation deficits (Imgrund et al., 2009; Ginkel et al., 2012; Ebel et al., 2013). Perhaps findings observed in first-episode SZ patients with lower levels of ceramide 1 (a long chain ceramide) and higher levels of ceramides 5 and 8 (short chain ceramides) may be explained in this context (Smesny et al., 2013).

**Figure 5:**
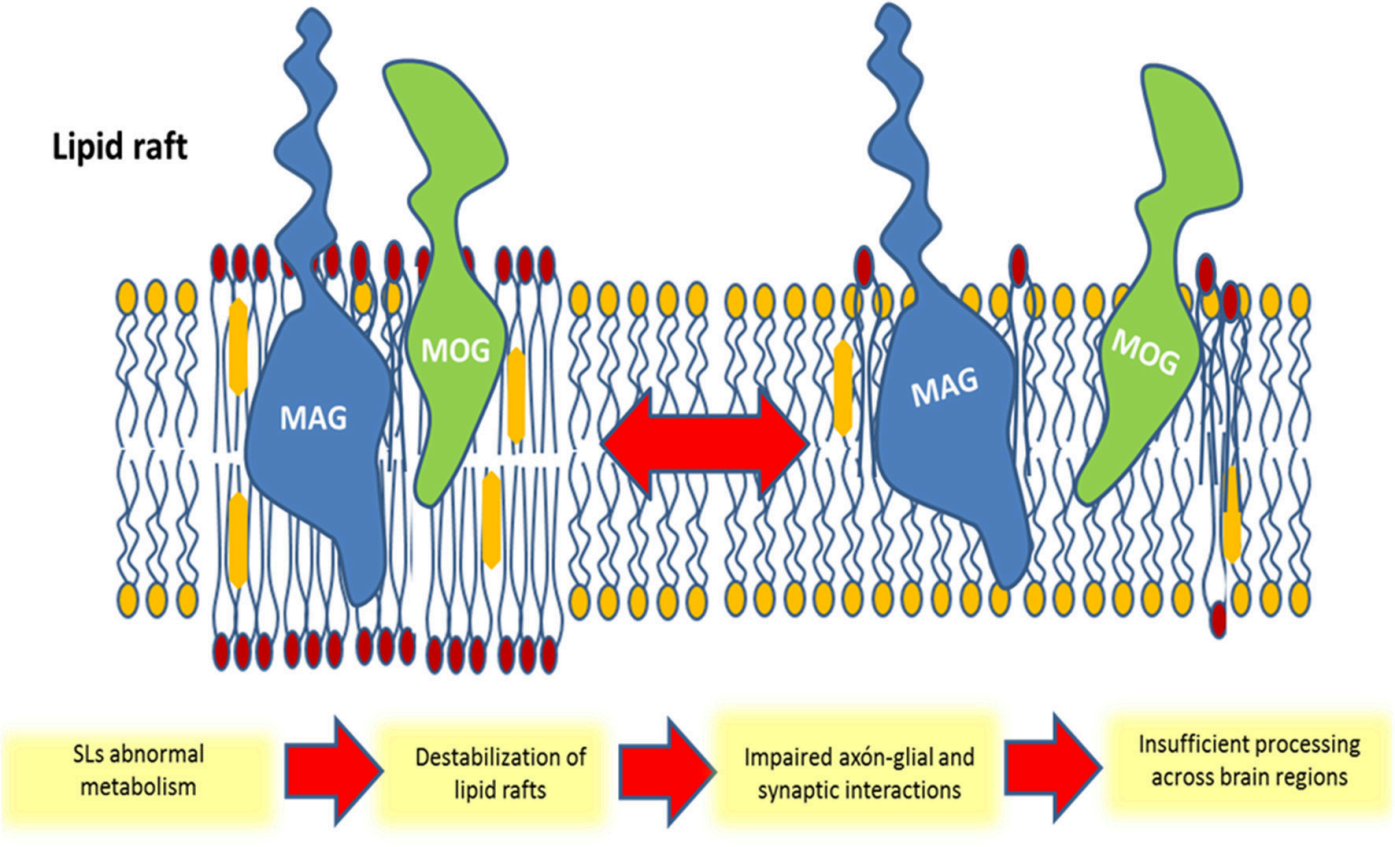
**Schematic representation of the participation of membrane glycosphingolipid-enriched microdomains in schizophrenia**. The repartitioning of molecules into (or out of) lipid rafts can lead to an impaired myelin structure in oligodendrocytes and impaired synaptic connectivity in neurons. In this example, oligodendrocytes myelin-associated glycoprotein (MAG) and myelin/oligodendrocyte glycoprotein (MOG) are segregated from the lipid raft following a structural sphingolipid abnormality, leading to an insufficient axonal-glial interaction and loss of brain connectivity.

One potential explanation for these behavioral consequences is a membrane microdomain destabilization due to a lack of structural SLs. As suggested above, membrane “lipid rafts” are mostly formed and regulated by SLs. This is important for axonal-glial (Figure 5) and synaptic interactions, both crucial for proper brain connectivity (Jackman et al., 2009). In this context, the study by Colón-Sáez and Yakel reported that the enzymatic hydrolysis of SMs in rat hippocampal neurons alters the α-7 nicotinic acetylcholine receptor function, presumably influencing its anchoring in the lipid rafts (Colón-Sáez and Yakel, 2011).

Overall, the discussed evidence supports the hypothesis that SZ disconnectivity can be explained by alterations in membrane SLs metabolism, which lead to a decreased axonal myelination and impaired synaptic plasticity (Figure 5).

### Future of sphingolipids in the development of new diagnostic and therapeutic technologies for schizophrenia-related metabolic syndrome

For a long time, clinicians have speculated about the potential options to exert therapeutic interventions during the early stages of SZ, with the goal of delaying or averting the illness and/or its complications (Sullivan, 1994; Srihari et al., 2013). However, effective early interventions require the ability to correctly identify the patients that will have a poor clinical course and more comorbid conditions, like MS.

Even though international clinical guidelines recommend undertaking a complete cardiovascular risk assessment in first episode psychosis patients before pharmacological treatment is initiated (De-Hert et al., 2009), the routine metabolic screening at early stages does not consider a MS genetic vulnerability. In fact, we do not have clinically validated biomarkers for the diagnosis or follow up of SZ, nor its metabolic complications (Weickert et al., 2013). Thus, it is still a challenge to select a particular subpopulation of SZ patients to follow.

Emerging evidence suggests that SLs may be suitable biomarkers for MS, revealing important associations between MS and the level of certain SLs in peripheral blood samples. There are at least six studies that show elevated levels of ceramides or GM3 gangliosides in overfed, overweight or diabetic/dyslipidemic patients in peripheral samples. Additionally, these abnormalities are usually correlated with elevation in other parameters, such as adiponectin, cholesterol, TNFα and glucose (Sato et al., 2008; Majumdar and Mastrandrea, 2012; Heilbronn et al., 2013; Lopez et al., 2013; Ng et al., 2015; Veillon et al., 2015) (Table 1). These results are paralleled by similar findings in an overfed and diabetic primate model (Brozinick et al., 2013).

**Table 1:** **Sphingolipids, and other related lipids in peripheral samples as biomarkers of schizophrenia or metabolic syndrome in humans**.

**References**	**N**	**Subjects**	**Gender**	**Age**	**Sample**	**Main Findings**
**SCHIZOPHRENIA STUDIES**						
Schwarz et al., 2008	20	First episode and chronic	Both	36.85 ± 8.4	Blood (red blood cells)	↑Cer 34:1 and stearic acid under SGA treatment.
Smesny et al., 2013	28	First episode	Both	23.27 ± 3.6	Skin (stratum corneum)	↓Total Cer, ↑Cer AH and NH/AS, ↓Cer EOS and NP.
He et al., 2012	265	Chronic	Both	19–67	Blood (Plasma)	↓PC C38:6.
**METABOLIC SYNDROME STUDIES**						
Heilbronn et al., 2013	40	Overfed	Both	37 ± 2	Blood	↑Total Cer, C22:0 and C24:0 (correlated with LDL).
Lopez et al., 2013	14	DM2	Female	14.3 ± 1.8	Blood (Plasma)	↑Cer C22:0, C20:0, C18:0, and C24:1 DihydroCer (correlated with adiponectin, HOMA-IR, BMI, fasting glucose, TG).
Majumdar and Mastrandrea, 2012	30	Overweight	Both	14.6 ± 1.2	Blood (Serum)	↑Cer correlated with TNF-*α*, adiponectin, lipoproteins, and HOMA-IR levels.
Ng et al., 2015	12	MS	Men	48.6 ± 8.5	Blood (Plasma)	VLDL apoB-100 directly correlated with longer chain Cer concentrations (C20:0, C22:0, C24:1, C24:0).
Veillon et al., 2015	39	Visceral fat ± hyperglycemia/dyslipidemia	Both	52.1 ± 1.5	Blood (Serum)	↑GM3 species. GM3 d18:1-h24:1 was the best candidate for metabolic screening.
Sato et al., 2008	55	DM2 and hyperlipidemic	Both	31–84	Blood (Serum)	↑GM3 species. GM3 was directly correlated with LDL.

*↓, decreased levels; ↑, increased levels; Cer, ceramides; MS, metabolic syndrome; AH, α-hydroxy 6-hydroxysphingosine; NH, nonhydroxy 6-hydroxysphingosine; AS, α-hydroxy sphingosine; EOS, ω-hydroxysphingosine; NP, nonhydroxy phytosphingosine; HOMA-IR, Insulin resistance homeostasis model assessment; TNF-α, tumor necrosis factor α; PC, phosphatidylcholine; SGA, second generation antipsychotics; DM, diabetes mellitus; TG, triglycerides; GM3, ganglioside GM3; LDL, low density lipoprotein; VLDL, very low density lipoprotein; BMI, body mass index*.

There is also evidence showing association between SZ and SLs levels in peripheral samples (Table 1). Schwarz et al. demonstrated elevations of specific ceramides (C34:1) and FFAs (stearic acid) in peripheral red blood cell samples from SZ patients after using second-generation antipsychotics (Schwarz et al., 2008). The study of Smesny et al. (See Table 1) supports a SZ-SLs direct association, since they found SLs abnormalities in the stratum corneum of the skin of first-episode drug-naïve SZ patients, representing a novel and non-invasive test for psychiatric diagnosis (Smesny et al., 2013). The rationale behind this approach is the large representation and structural homology between skin and CNS lipids. In this case, the researchers found only ceramides abnormalities among different classes of skin lipids. Lastly, other studies have found decreased PC, which might be related to increase SMs breakdown and thus increased ceramides levels in SZ patients (Brozinick et al., 2013).

Whether the association between SLs and SZ is related to only a specific subpopulation of SZ patients at risk to develop MS is still unknown, and future longitudinal studies are necessary in this area. Due to the importance of SLs metabolism in neurodevelopment and in general lipid homeostasis, it is also necessary to explore whether SLs alterations in MS and SZ are indicating a common pathophysiological alteration with different phenotypic manifestations. In this regard, it will be important to recruit SZ patients without central obesity for new studies, so as to demonstrate that metabolic disturbances are independent from classical MS mechanisms in these patients.

Although the presented studies have diverging purposes, subjects and results, there are relevant common conclusions, which can support the relationships between SZ and SLs metabolism. First, it is possible to measure significant alterations in SLs in peripheral samples like blood and skin cells, which may reflect actual SLs alterations in CNS and other tissues. This is important since it opens the possibility for developing an ideal non-invasive biomarker for a SNC condition. Second, although it is still necessary to have more compelling evidence with proper controls for all MS risk factors, current literature suggest that MS is also present in young antipsychotics-free populations regardless of disease progression and pharmacological treatment. Finally, although the current studies have focused on the analyzes of different subtypes of lipids in biological samples, which make data comparability rather difficult, all of them suggest that the inflammatory/structural ratio of SLs ratio is increased, as a common metabolic feature of SZ and MS. This is interesting if we consider that both, SZ and MS, correspond to complex polygenic alterations and gene-environment interactions. Perhaps a hypo/hyperfunction of some SLs-related enzymes may trigger these abnormalities.

Consequently, we propose the testable hypothesis that a specific SLs profile from peripheral tissues will serve as a MS biomarker to diagnose and evaluate the natural evolution of MS in psychotic patients. Since SZ is a highly heterogenic disease, this biomarker should allow us to recognize a differential vulnerability for metabolic disturbances among SZ patients. In this context, prodromal and first-episode psychosis populations represent a good and early opportunity to make a complete evaluation in order to target specific preventive interventions for these patients.

Finally, it is worth mentioning in this review that many of the emerging alternative treatments proposed for SZ [e.g., vitamins (Arroll et al., 2014; Brown and Roffman, 2014), omega-3 fatty acids (Hashimoto et al., 2014), anti-inflammatory agents (Girgis et al., 2014)] can have direct and/or indirect impacts on inflammatory status, membrane lipids profiles, and membrane structural properties (Yaqoob and Shaikh, 2010). Omega-3 fatty acids in particular have important effects on brain functions since docosahexaenoic acid (DHA) is a major structural component of phospholipids in neuronal cell membranes and eicosapentaenoic acid (EPA) shows neurotransmitter and neuromodulatory activity (Fenton et al., 2000) which may have therapeutic properties in psychotic symptoms. In fact, recent evidence has confirmed that young adults with sub-threshold psychotic symptoms lower their risk of developing a psychotic disorder when treated for 12 weeks with a formulation containing omega-3 essential polyunsaturated fatty acids (PUFAs), EPA, DHA, and tocopherol (Vitamin E) (Amminger et al., 2010, 2015). This opens new possibilities to investigate the specific role of SLs metabolism in SZ, with the goal of finding new therapeutic opportunities in SZ.

## Conclusions

**Figure 6:**
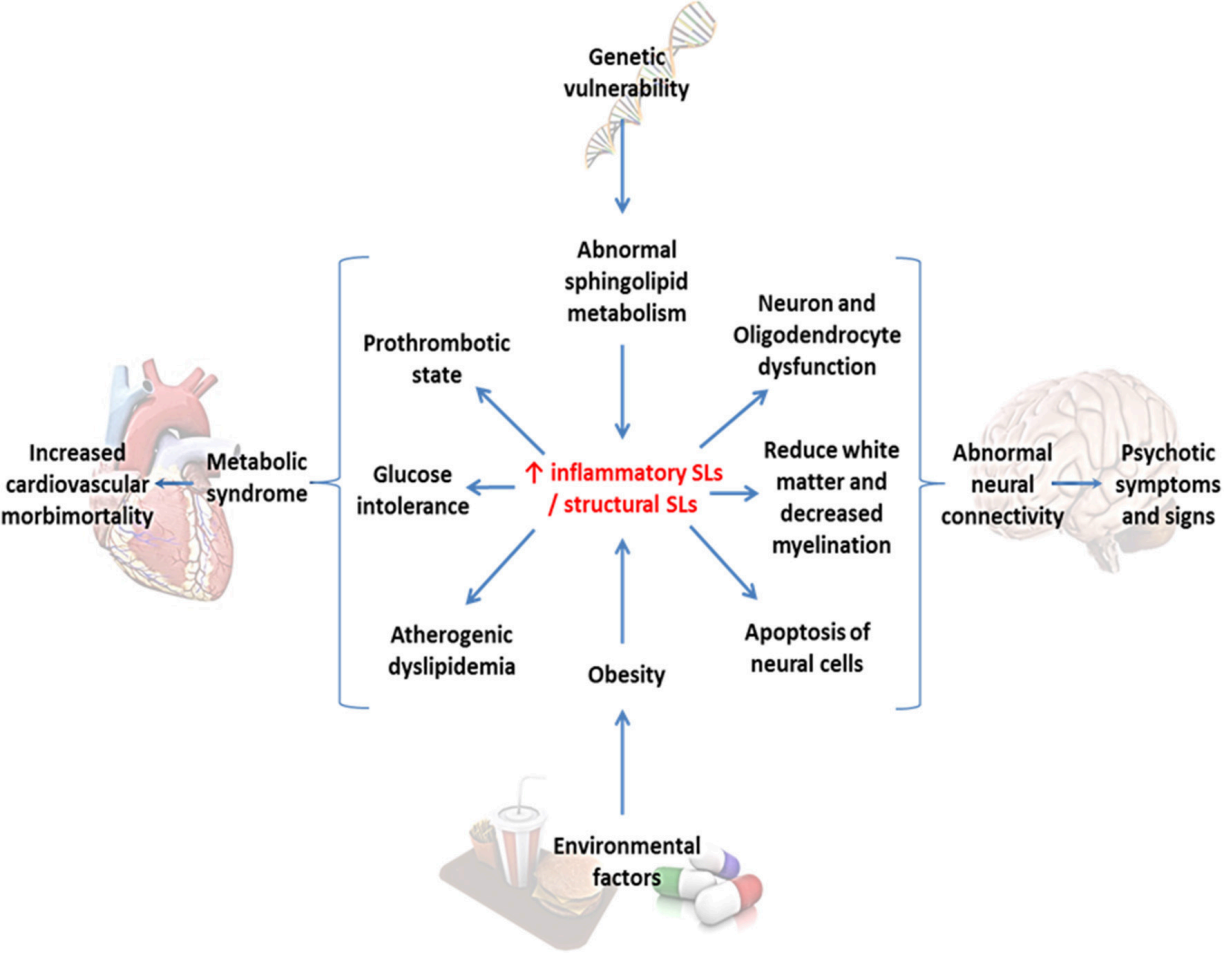
**Sphingolipids as the central convergence point between metabolic syndrome and schizophrenia**. Environmental factors and intrinsic genetic vulnerability converge in an abnormal sphingolipid metabolism, in this case represented in an increased inflammatory/structural sphingolipid ratio. This alteration might lead to both metabolic and neuronal abnormalities secondarily provoking cardiovascular morbidity and psychotic symptoms.

In summary, we have reviewed the evidence linking SLs, MS, and SZ, hinting a plausible common etiopathogenic a mechanism, which explains the clinical features like the vulnerability of these patients to develop metabolic complications, regardless of the presence of antipsychotic drugs, at early stages of the disease. Moreover, dysfunction in SLs pathway may represent an important connecting-piece in understanding the SZ neurobiology, allowing us to link the current diverse theories of SZ pathology (Figure 6).

As suggested above, measuring specific levels of SLs in biological samples and defining a specific profile of these molecules in skin tests or in peripheral blood samples could help us to anticipate cardiovascular complications and/or to make an early SZ diagnosis, especially in the group of patients at prodromal and first episode of psychosis. By using SLs profiles as MS and/or SZ biomarkers in this population, we could develop a more personalized clinical management to improve the patient's prognosis. Finally, this association opens new possibilities for innovative research in the SLs field toward finding novel pharmacological treatments such as omega-3 fatty acids, which may impact both psychotic and/or metabolic symptoms.

Future studies should confirm these statements in order to find the most representative profile of SLs involved in SZ and to prove the feasibility and clinical utility of these examinations.

## Author contributions

RC, PG, and LR conceived and outlined the manuscript. RC wrote the manuscript under the supervision of PG and LR. PG contributed with biological and clinical aspects of schizophrenia. LR contributed to describe the links between metabolic syndrome and sphingolipid metabolism (metabolic pathways, signal transductions, etc.). MH contributed with molecular aspects of lipids metabolism in the context of brain physiology. HS edited the entire manuscript and wrote the section on development of future therapeutic technologies. AM contributed to edit the manuscript and wrote the section “Sphingolipids and SZ disconnectivity hypothesis.” MV contributed with the sphingolipds and metabolic syndrome. MF outlined and wrote the relationships between sphingolipids and schizophrenia.

## Funding

RC is supported by CONICYT-PCHA, Doctorado Nacional, 2015-21150063, Ministry of Education, Chile. PG is funded by the National Commission for Scientific and Technological Research (CONICYT), FONDECYT initiation into research 2014, grant No. 11140464 and OAIC grant from the Clinical Hospital of the University of Chile. The Biomedical Neuroscience Institute (BNI) supports PG and HS. LR is funded by the National Commission for Scientific and Technological Research (CONICYT), FONDECYT initiation into research 2014, grant No. 11140915.

### Conflict of interest statement

The authors declare that the research was conducted in the absence of any commercial or financial relationships that could be construed as a potential conflict of interest. The reviewer JN and handling Editor declared their shared affiliation, and the handling Editor states that the process nevertheless met the standards of a fair and objective review.

## Nomenclature

ADRA1A = Alpha-1A adrenergic receptor

Akt/PKB = Protein Kinase B

BDNF = Brain-derived neurotrophic factor

CerS = Ceramide Synthase

CGT = Galactosyltransferase

CNS = Central Nervous System

CST = Cerebroside Sulfotransferase

DHA = Docosahexaenoic acid

DTI = Diffusion Tensor Imaging

EEG = Electroencephalogram

EPA = Eicosapentaenoic acid

FFAs = Free Fatty Acids

fMRI = Functional Magnetic Resonance Imaging

GABA = Gamma-Aminobutyric Acid

GLUT-4 = Glucose Transporter Type 4

GSLs = Glycosphingolipids

HDL = High Density Lipoprotein

IRS-1 = Insulin Receptor Substrate-1

IκKβ = Inhibitor of kappa B Kinase beta

JNK = c-Jun N-Terminal Kinases

LDL = Low Density Lipoprotein

MS = Metabolic Syndrome

MTHFR = Methylenetetrahydrofolate Reductase

NMDA = N-methyl-D-aspartate

PAI-1 = Plasminogen Activator Inhibitor-1

PC = Phosphatidylcholine

PI3K = Phosphoinositide 3-kinase

PKCζ = Protein Kinase C zeta

PP2A = Protein Phosphatase 2A

PUFAs = Polyunsaturated fatty acids

S1P = Sphingosine-1-Phosphate

SFAs = Saturated Fatty Acids

SLs = Sphingolipids

SMs = Sphingomyelins

SREBPs = Sterol-Regulatory Element Binding Proteins

SZ = Schizophrenia

TNF-α = Tumor Necrosis Factor α
